# Incidentally Identified Pneumatosis Cystoides Intestinalis in Patients With Gastric Outlet Obstruction From Addis Ababa, Ethiopia: A Case Report

**DOI:** 10.1155/cris/9963907

**Published:** 2026-05-30

**Authors:** Megersa Regassa, Terefe Meshesha, Kaleb Assefa Berhane, Meron Zeleke

**Affiliations:** ^1^ Department of Surgery, Adera Medical and Surgical Center, Addis Ababa, Ethiopia; ^2^ Department of Surgery, Yekatit 12 Hospital Medical College, Addis Ababa, Ethiopia; ^3^ Department of General Medicine, Adera Medical and Surgical Center, Addis Ababa, Ethiopia

**Keywords:** case report, Ethiopia, gastric outlet obstruction, peptic ulcer disease, pneumatosis cystoides intestinalis

## Abstract

Pneumatosis cystoides intestinalis (PCI) is a rare condition characterized by the presence of multiple gas‐filled cysts within the submucosal or subserosal layers of the intestinal wall. Although often asymptomatic and detected incidentally, PCI may be associated with significant underlying gastrointestinal or systemic pathology. We report two young adults from Addis Ababa, Ethiopia, who presented with long‐standing upper gastrointestinal symptoms, including early satiety, postprandial epigastric pain, reflux, vomiting, and marked weight loss. Clinical, laboratory, and endoscopic evaluations confirmed gastric outlet obstruction (GOO) secondary to chronic duodenal stenosis. Both patients underwent retrocolic gastrojejunostomy with truncal vagotomy and Braun’s anastomosis. During surgery, multiple gas‐filled cystic lesions consistent with PCI were incidentally identified on the serosal surface of the small intestine. There were no signs of bowel ischemia, perforation, or peritonitis. No bowel resection was performed, and PCI was managed conservatively. Postoperative recovery was uneventful in both cases, with complete symptom resolution and significant weight gain at follow‐up. These cases emphasize that PCI may occur secondary to chronic mucosal disruption and increased intraluminal pressure in GOO. Recognition of PCI in this clinical context is essential to guide appropriate management. When incidentally detected in the absence of complications, PCI does not warrant specific surgical intervention. Management should instead focus on addressing the underlying pathology to avoid unnecessary bowel resection.

## 1. Introduction

**Figure 1:**
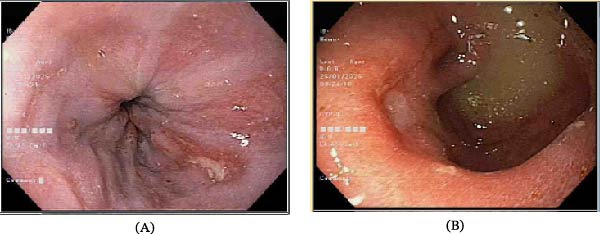
Case 1: (A) Los Angeles grade B esophagitis. (B) Endoscopic view showing severe pyloric stenosis with retained gastric contents, consistent with GOO, along with a duodenal ulcer and pseudodiverticulum.

**Figure 2:**
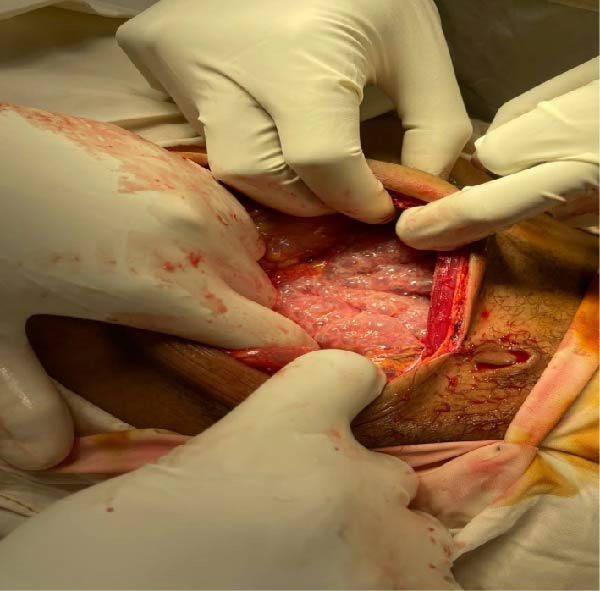
Case 1: Intraoperative image showing the initial exposure of the abdominal cavity, revealing distended bowel loops and serosal gas‐filled cysts suggestive of PCI.

Pneumatosis cystoides intestinalis (PCI) is a condition marked by the presence of gas‐filled cysts within the submucosal and subserosal layers of the intestinal wall [1]. It was first identified in 1730 by DuVernoi during a cadaver dissection. The first radiologic diagnosis was made in 1946 by Lerner and Gazin. Although the number of reported cases has increased with advancements in modern imaging, PCI still remains rare, with an estimated prevalence of about 0.03% in the general population. However, due to the predominantly asymptomatic nature of the condition, its actual prevalence remains unclear, and the reported rate (0.03%) is likely lower than the true figure [1, 2].

The pathophysiology of PCI also remains unclear; however, several contributing factors have been implicated, including intestinal barrier dysfunction, microbial involvement, and respiratory disorders. PCI is often a rare incidental finding, typically discovered by radiologists in asymptomatic patients. Nevertheless, it can also be an indicator of serious underlying conditions such as intestinal obstruction, bowel ischemia, or mesenteric vascular accidents, which may be life‐threatening [2].

Most cases of PCI are benign and can be managed conservatively. However, in the presence of complications such as bowel ischemia, perforation, or portal venous gas, surgical intervention may be necessary [3]. We present a case report of two patients with gastric outlet obstruction (GOO) secondary to pyloric stenosis, in whom PCI was incidentally discovered during surgery. This case report was prepared following the guidelines of the SCARE checklist [4].

## 2. Case 1

A 30‐year‐old male from Addis Ababa presented with a 3‐year history of progressively worsening postprandial epigastric pain, early satiety, bloating, belching, intermittent nausea, poor appetite, and an unintentional weight loss of 10 kg over the past year. He reported self‐induced vomiting for symptom relief. Although he initially responded to proton pump inhibitors (PPIs), his symptoms later became refractory, affecting his sleep and causing significant psychological distress. The patient had a history of chronic khat chewing and cigarette smoking, both of which he discontinued 3 months prior to presentation. He denied alcohol use and had no significant past medical or family history.

On physical examination, the patient appeared slim but alert and in no acute distress. He was hemodynamically stable, with vital signs within normal limits. Abdominal examination revealed a scaphoid abdomen with mild epigastric tenderness and a positive succussion splash. There were no palpable masses or organomegaly.

Laboratory findings, including complete blood count (CBC), renal and liver function tests, and serum electrolytes, were within normal limits. Fecal occult blood and stool *H. pylori* tests were negative. Abdominal ultrasonography showed a markedly dilated stomach. Esophagogastroduodenoscopy (EGD) revealed Los Angeles grade B gastroesophageal reflux disease (GERD), multiple erosions at the pylorus, a superficial 5 mm antral ulcer, and a markedly dilated, adynamic stomach containing a pool of grayish, turbid food and fluid. There was severe pyloric stenosis (Figure 1).

A diagnosis of GOO was made, and the patient was taken to the operating room for exploratory laparotomy under general anesthesia.

Intraoperatively, multiple gas‐filled cystic lesions were observed on the serosal surface of the small intestine, sparing only the proximal jejunum. These lesions were multilobulated, translucent, and measured between 0.5 and 2.5 cm in diameter, creating a characteristic “grape‐like” or “bubbly” appearance (Figure 2). There were no signs of bowel ischemia, perforation, or peritonitis.

Moreover, a cicatrized and stenosed pylorus, first part of the duodenum, and hugely dilated stomach were noted, and the patient underwent a retrocolic gastrojejunostomy with truncal vagotomy and Braun’s anastomosis. Postoperative recovery was uneventful. By the third postoperative day, he had resumed oral intake with improved appetite and gradual resolution of symptoms. Follow‐up at 6 months postoperatively showed no recurrence of symptoms and notable weight gain.

## 3. Case 2

A 28‐year‐old woman from Addis Ababa, Ethiopia, presented with a 5‐year history of progressively worsening postprandial bloating, nausea, and occasional nonbilious vomiting. Initially intermittent, the symptoms became near‐daily, with marked postmeal discomfort, indigestion, upper abdominal pressure, and acid reflux, particularly at night. Over the past 6 months, she experienced an unintended 7 kg weight loss. She also reported sleep disturbances from nighttime reflux but denied hematemesis or melena. There was no family history of gastrointestinal disease, prior abdominal surgery, or substance use. Multiple short courses of PPIs offered only transient and partial symptom relief.

On physical examination, the patient appeared underweight but was alert and oriented. She was hemodynamically stable, afebrile, and mildly dehydrated. Abdominal examination revealed mild distension, tenderness in the epigastrium, and a positive succussion splash. No guarding, rebound, or masses were detected.

**Figure 3:**
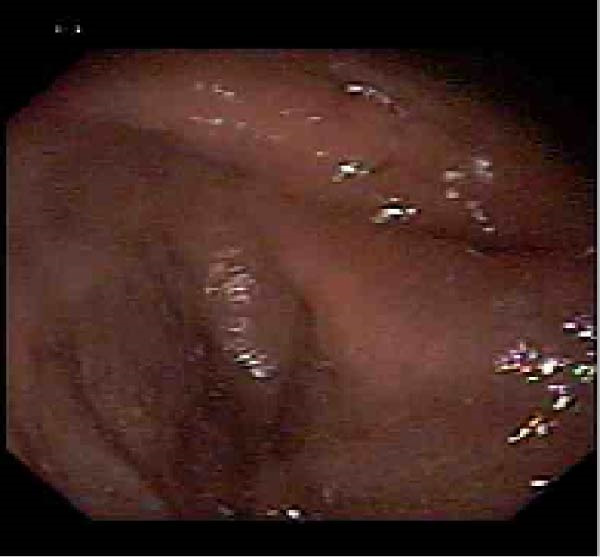
Case 2: Endoscopic view showing deformity and narrowing of the duodenal bulb suggestive of gastric outlet obstruction.

Laboratory investigations, including CBC, renal function tests, liver enzymes, and serum electrolytes, were all within normal limits. Abdominal ultrasound demonstrated a distended stomach with delayed emptying. EGD revealed Los Angeles grade A reflux esophagitis, inflammatory changes in the antrum, and a narrowed duodenal bulb with ulceration and scarring (Figure 3).

**Figure 4:**
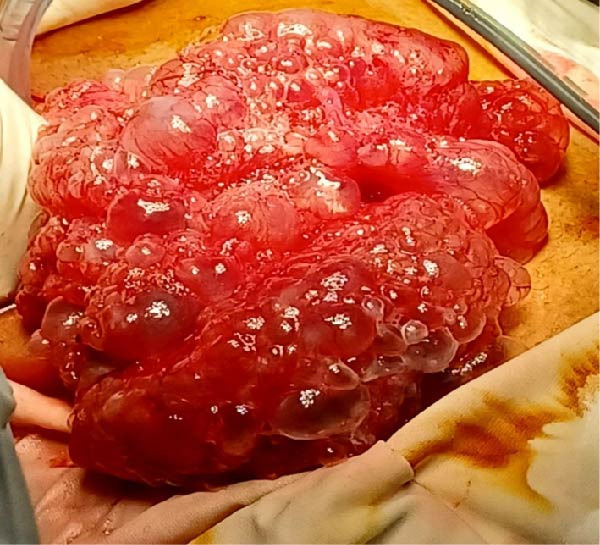
Case 2: Intraoperative view of the small intestine showing numerous translucent, gas‐filled cystic lesions along the serosal surface, characteristic of PCI.

At laparotomy, the stomach was markedly dilated. Incidentally, numerous gas‐filled cystic structures were observed on the serosal surface of the whole small bowel. These cysts were irregularly distributed, measuring between 0.3 and 5 cm in diameter, giving the bowel a finely nodular, “soap‐bubble” appearance (Figure 4). There were no signs of inflammation, ischemia, or bowel compromise.

The patient underwent a retrocolic gastrojejunostomy, truncal vagotomy, and Braun’s anastomosis to address the GOO. Her postoperative course was smooth, with oral intake resumed on the third day and gradual symptom improvement. At follow‐ups at 2 weeks, 2 months, and 6 months postsurgery, she reported complete resolution of symptoms, improved nutritional intake, and a weight gain of 5 kg, with no evidence of recurrence or complications.

## 4. Discussion

PCI, also known as pneumatosis intestinalis, intraluminal bowel gas, or pneumatosis coli—depending on the location and pattern of gas accumulation—refers to the presence of gas outside the intestinal lumen. Specifically, PCI involves multiple gas‐filled cysts forming within the submucosal and subserosal layers of the intestine. If not treated, these cysts can grow in both number and size. PCI can affect individuals of all ages, though it is more commonly observed in older adults than in young adults or infants [2]. The male‐to‐female ratio varies across studies. A systematic review of 77 reports involving 239 cases found a male predominance with a ratio of 2.4:1, while other studies have reported equal prevalence between genders [5]. In our own case report, we reported one male and one female young adult patient.

PCI can be classified as primary (15%) and secondary (85%). Primary PCI is typically benign, with gas forming cyst‐like patterns in the intestinal wall. In contrast, secondary PCI is linked to underlying diseases and presents with a more linear gas distribution. It is associated with gastrointestinal disorders such as IBD, diverticular disease, ischemic and pseudomembranous colitis, Hirschsprung’s disease, sigmoid volvulus, pyloric stenosis, and surgical anastomoses, as well as non‐GI conditions like collagen vascular diseases, chronic obstructive pulmonary disease, asthma, and cystic fibrosis [2, 6]. In this case report, the underlying pathology leading to PCI was pyloric stenosis secondary to long‐standing peptic ulcer disease, resulting in GOO. So far, only two prior cases of PCI associated with GOO have been reported from Ethiopia [6, 7].

Various imaging modalities can aid in the diagnosis of PCI, including plain abdominal radiography, ultrasonography, computed tomography (CT), and magnetic resonance imaging (MRI). However, one‐third of patients’ radiographs may not reveal any signs, such as thickened bowel walls containing gas. CT is the most sensitive imaging modality, as it better distinguishes intraluminal air from submucosal fat and detects additional underlying causes of PCI such as portal air, colonic tissue stranding, and dilated bowel. Serial imaging may be warranted to monitor progression and to evaluate for the development or extension of portal venous or intra‐abdominal free air [2].

Three main theories have been proposed to explain the pathogenesis of PCI. The mechanical theory suggests that increased intraluminal pressure leads to mucosal disruption, permitting gas to dissect into the bowel wall. The pulmonary theory posits that alveolar rupture, often due to chronic lung disease, allows gas to travel along the mediastinum and retroperitoneum into the intestinal wall. Last, the bacterial theory implicates gas‐producing organisms that penetrate the mucosa and release gas within the bowel wall [2, 6, 8]. Our case report aligns with the mucosal disruption theory, which attributes the presence of intramural gas to mechanical breakdown of the gastrointestinal mucosa.

Pneumatosis cysts may develop at any site within the gastrointestinal tract, with the large intestine (46%), small intestine (27%), stomach (5%), and combined locations (7%) being the most frequently reported. Despite this broad distribution, the terminal ileum remains the most common site of involvement. Rarely, cysts have also been detected in atypical locations such as the mesentery, omentum, and ligamentum hepatogastricum [9]. In our case, PCI was observed to be limited the most parts of the small intestine.

PCI presents with a wide range of clinical manifestations, from asymptomatic cases to nonspecific gastrointestinal symptoms such as diarrhea, abdominal distension, weight loss, and the passage of blood or mucus in the stool. While these symptoms are often nonspecific, they generally correspond to the anatomical site of involvement and any underlying pathology. Patients with conditions like pyloric stenosis or peptic ulcer disease typically exhibit upper gastrointestinal symptoms, whereas involvement of the colon is more likely to produce lower gastrointestinal signs such as diarrhea, bloody stools, and marked abdominal distension [6, 9]. In our report, the patients presented with upper gastrointestinal symptoms consistent with features of GOO accompanied by significant weight loss. A previously reported case from Ethiopia described a 44‐year‐old man with chronic vomiting and epigastric pain who was diagnosed with GOO, in whom PCI was discovered incidentally during surgery [7]. In another report, Bayissa et al. [6] documented a 25‐year‐old patient presenting with persistent vomiting, intermittent abdominal cramps, and significant weight loss, who was found to have GOO accompanied by extensive small bowel PCI.

Comparative studies suggest that conservative management, including observation, hyperbaric oxygen therapy, antibiotics to address intestinal bacterial infection, and endoscopic monitoring, is effective in the short term and is generally recommended, accompanied by ongoing imaging and endoscopic surveillance. In our cases, both patients had longstanding symptoms suggestive of chronic peptic ulcer disease. Delayed presentation and prolonged mucosal injury likely resulted in fibrotic pyloric and duodenal stenosis. Once structural narrowing develops, medical therapy alone (e.g., PPIs) is unlikely to reverse obstruction. Intermittent or incomplete adherence to therapy may also have contributed. Although the definitive treatment of PCI depends on the underlying etiology, early surgical intervention in a hemodynamically stable patient without a trial of conservative therapy may lead to unfavorable outcomes. Surgical management is typically reserved for complications such as intestinal obstruction, perforation, or suspected malignancy [2, 10]. In our current cases, surgery was indicated due to coexisting GOO caused by duodenal stenosis. Bowel resection was not performed, as intraoperative findings revealed no compromise in bowel viability and no signs of perforation. The surgical approach was thus directed at relieving the obstruction to manage the primary pathology and mitigate factors contributing to the secondary pneumatosis intestinalis. The stable postoperative recovery further supports the benign nature of PCI in the absence of features suggestive of ischemia or perforation. Postoperative imaging specifically evaluating PCI was not performed, as both patients remained clinically stable without signs suggestive of progression or complications.

These cases highlight the importance of recognizing PCI as a rare but possible incidental finding during surgery for gastrointestinal conditions such as GOO. In the absence of bowel ischemia, perforation, or peritonitis, PCI does not warrant direct surgical intervention. Management should instead focus on treating the underlying pathology. Awareness of PCI’s typically benign course in such contexts helps avoid unnecessary bowel resections and guides appropriate surgical decision‐making.

**Nomenclature**
CBC: Complete blood count
EGD: Esophagogastroduodenoscopy
GERD: Gastroesophageal reflux disease
GOO: Gastric outlet obstruction
PCI: Pneumatosis cystoides intestinalis
PPI: Proton pump inhibitor.

## Author Contributions

**Megersa Regassa:** conceptualization, data curation, investigation, resources, writing – review and editing. **Terefe Meshesha**: conceptualization, methodology, visualization, investigation, writing – review and editing. **Kaleb Assefa Berhane**: conceptualization, project administration, writing – original draft, writing – review and editing. **Meron Zeleke**: conceptualization, writing – original draft, writing – review and editing.

## Funding

No funding was received for this research.

## Disclosure

All authors reviewed and approved the final version of the manuscript.

## Ethics Statement

The study is exempt from ethical approval at our institution. All the information obtained was held confidential and used only for the intended purpose.

## Consent

Written informed consent was obtained from the patient for publication of this case report and the accompanying images.

## Conflicts of Interest

The authors declare no conflicts of interest.

## Data Availability

The data that support the findings of this study are available from the corresponding author upon reasonable request.

## References

[1] Wang Y. J. , Wang Y. M. , Zheng Y. M. , Jiang H. Q. , and Zhang J. , Pneumatosis Cystoides Intestinalis: Six Case Reports and a Review of the Literature, BMC Gastroenterology. (2018) 18, no. 1, 10.1186/s12876-018-0794-y, 2-s2.0-85049157158, 100. 29954324 PMC6022295

[2] Im J. and Anjum F. , Pneumatosis Intestinalis, 2025, StatPearls Publishing, https://www.ncbi.nlm.nih.gov/books/NBK564381/. 33232051

[3] Saber A. , Pneumatosis Intestinalis With Complete Remission: A Case Report, Cases Journal. (2009) 2, no. 1, 10.1186/1757-1626-2-7079, 2-s2.0-77953366702, 7079. 20184685 PMC2827120

[4] Sohrabi C. , Mathew G. , Maria N. , Kerwan A. , Franchi T. , and Agha R. A. , The SCARE 2023 Guideline: Updating Consensus Surgical CAse REport (SCARE) Guidelines, International Journal of Surgery. (2023) 109, no. 5, 1136–1140, 10.1097/JS9.0000000000000373. 37013953 PMC10389401

[5] Wu L. L. , Yang Y. S. , Dou Y. , and Liu Q. S. , A Systematic Analysis of Pneumatosis Cystoids Intestinalis, World Journal of Gastroenterology. (2013) 19, no. 30, 4973–4978, 10.3748/wjg.v19.i30.4973, 2-s2.0-84881524892. 23946603 PMC3740428

[6] Bayissa B. B. , Fanos B. , Tufa D. W. , Admasu B. , Alemu A. , and Getiye A. , Extensive Inflammatory Adhesion of Small Bowel With Massive Pneumatosis Intestinalis in a Patient With Gastric Outlet Obstruction: A Rare Case Report, International Journal of Surgery Case Reports. (2024) 122, no. C, 10.1016/j.ijscr.2024.110152, 110152. 39154563 PMC11378174

[7] Nureta T. H. , Moges T. G. , and Abebe D. M. , Pneumatosis Cystoides Intestinalis Associated With Gastric Outlet Obstruction; A Case Report, International Journal of Surgery Case Reports. (2023) 111, 10.1016/j.ijscr.2023.108828, 108828. 37716064 PMC10509697

[8] Ling F. , Guo D. , and Zhu L. , Pneumatosis Cystoides Intestinalis: A Case Report and Literature Review, BMC Gastroenterology. (2019) 19, no. 1, 10.1186/s12876-019-1087-9, 176. 31694581 PMC6836417

[9] Moyon F. X. , Molina G. A. , and Tufiño J. F. , et al.Pneumoperitoneum and Pneumatosis Cystoides Intestinalis, a Dangerous Mixture. A Case Report, International Journal of Surgery Case Reports. (2020) 74, no. C, 222–225, 10.1016/j.ijscr.2020.07.086. 32892124 PMC7484497

[10] Ebrahimian M. , Ghayebi N. , and Rezaee S. P. , Gastric Pneumatosis and Concurrent Aeroportia Due to Gastric Outlet Obstruction: A Case Report, International Journal of Surgery Case Reports. (2021) 89, no. C, 10.1016/j.ijscr.2021.106584, 106584. 34784530 PMC8599093

